# Programming an Artificial Neural Network Tool for Spatial Interpolation in GIS - A Case Study for Indoor Radio Wave Propagation of WLAN

**DOI:** 10.3390/s8095996

**Published:** 2008-09-25

**Authors:** Alper Şen, M. Ümit Gümüşay, Aktül Kavas, Umut Bulucu

**Affiliations:** 1 Geodesy and Photogrammetry Engineering Department, Yildiz Technical University, Besiktas-Istanbul, Turkey; E-Mail: alpersen79@hotmail.com; 2 Electronics and Communications Engineering Department, Yildiz Technical University, Besiktas-Istanbul, Turkey; E-mails: kavas@yildiz.edu.tr; ubulucu@hotmail.com

**Keywords:** WLAN, Electromagnetic coverage, GIS, Artificial neural networks, Kriging, Interpolation

## Abstract

Wireless communication networks offer subscribers the possibilities of free mobility and access to information anywhere at any time. Therefore, electromagnetic coverage calculations are important for wireless mobile communication systems, especially in Wireless Local Area Networks (WLANs). Before any propagation computation is performed, modeling of indoor radio wave propagation needs accurate geographical information in order to avoid the interruption of data transmissions. Geographic Information Systems (GIS) and spatial interpolation techniques are very efficient for performing indoor radio wave propagation modeling. This paper describes the spatial interpolation of electromagnetic field measurements using a feed-forward back-propagation neural network programmed as a tool in GIS. The accuracy of Artificial Neural Networks (ANN) and geostatistical Kriging were compared by adjusting procedures. The feedforward back-propagation ANN provides adequate accuracy for spatial interpolation, but the predictions of Kriging interpolation are more accurate than the selected ANN. The proposed GIS ensures indoor radio wave propagation model and electromagnetic coverage, the number, position and transmitter power of access points and electromagnetic radiation level. Pollution analysis in a given propagation environment was done and it was demonstrated that WLAN (2.4 GHz) electromagnetic coverage does not lead to any electromagnetic pollution due to the low power levels used. Example interpolated electromagnetic field values for WLAN system in a building of Yildiz Technical University, Turkey, were generated using the selected network architectures to illustrate the results with an ANN.

## 1. Introduction

The analysis of indoor radio propagation is essential for the maintenance of a sustainable and effective communication link in wireless systems. Sufficient radio propagation must be maintained to avoid the interruption of the data transmission, therefore, indoor radio propagation of wireless communication systems is modeled and analyzed by Artificial Neural Networks (ANN) and Kriging spatial interpolation methods in Geographic Information Systems (GIS). With the use of GIS model, an efficient wireless network can be designed.

The huge demand in the wireless industry has accelerated studies on the development of accurate indoor radio wave propagation prediction methods. Hence indoor radio wave propagation modeling is a quite new and still rapidly developing area and it has become essential with the installation of Wireless Local Area Networks (WLAN) inside buildings [1].

The indoor propagation channel differs considerably from the outdoor one. The distance between transmitter and receiver is shorter, due to high attenuation caused by the internal walls and furniture and often also because of the lower transmitter power. Before any propagation computation can be performed, these surfaces must be characterized. It appears that accurate geographical information of this nature must be obtained [2].

Many additional effects can occur within buildings, making indoor propagation a very complex and fascinating process, whose exact description in rigorous mathematic formulations is not feasible [1]. However, GIS including spatial interpolation patterns is very efficient for studying the radio wave propagation environment, the number, position and transmitter power of access points, electromagnetic coverage and the radiation level values.

Spatial interpolation is used to estimate values at locations within the area covered by existing observations and an important function of a GIS analysis. For this reason a wide variety of interpolation methods are practised and developed. Since it is essential to run an accurate interpolation method, several techniques based on data type are compared.

There are different classes of interpolation methods such as geometrical nearness (e.g. the Voronoi approach), statistical methods (e.g. natural neighbor interpolation, weighting inverse distances, Kriging), using basis functions (e.g. trend surface analysis, regularized smoothing spline with tension, method of local polynomials) and the Artificial Neural Networks (ANN) method [3].

Some comparisons between ANN and traditional interpolation methods were done in different studies. For example, ANN interpolation perfomed better than Kriging in predicting precipitation levels in high altitude regions [4]. Xingong Li reported the use of a feed-forward neural network for precipitation estimation and the neural network performed consistently well the interpolation in contrast to the other methods such as Voronoy cells, trend surface analysis, inverse distance weighted and ordinary Kriging [3, 5]. The difficulties that existing methods have to represent complex nonstationary relationships are listed by Rigol *et al.* [6]. Snell *et al.* used a multilayer feed-forward back-propagation ANN for the spatial interpolation of daily maximum surface air temperatures and in 94% of case comparisons, the predictive accuracy of the ANN was superior to the benchmark methods (spatial average, nearest neighbor and inverse distance methods) [7]. The numerical results, the advantages and drawbacks of ANN were also discussed by Bollivier *et al.* [8]. ANN models provided greater accuracy than the inverse distance and average methods for estimating daily weather variables [9]. The use of back propagating feed-forward multilayer ANN using a sigmoidal function produced significantly better results compared with other spatial interpolation methods and were better than linear and log-linear models [10]. However, Pariente maintained that Hopfield neural nets (Hopfield 84) were much more precise than feed-forward nets and the other interpolation methods [11].

Few studies have been done on the use of ANNs as tools for GIS and it is obvious that the future GIS implementations should have ANN modules and more research activity must be performed, especially for spatial interpolation [3, 12].

In this study, GIS was used to represent indoor radio wave propagation environment and electromagnetic coverage by means of ANN and Kriging interpolation patterns with geographical features. In order to illustrate the approach, electromagnetic field values were measured at the entrance floor of T-Block building where one of the wireless communication systems is available in Yildiz Technical University for analyzing indoor radio wave propagation of WLAN (2.4 GHz). The proposed GIS also ensures 3- dimensional modeling of the study area, the number, position and transmitter power of access points and electromagnetic radiation level.

The main goal of this paper is to integrate a multilayer feed-forward back-propagation ANN module in the GIS software (ArcGIS) by programming ArcObjects with Visual Basic to interpolate the indoor electromagnetic field measurements for propagation analysis. The accuracy was compared with geostatistical Kriging available in ArcGIS by adjusting procedures such as Root Mean Square Error and the Mean Absolute Error. It was demonstrated that the feed-forward back-propagation ANN for spatial interpolation of electromagnetic field measurements provides adequate accuracy. However, the predictions of Kriging interpolation are more accurate than the selected ANN model.

## 2. Literature Review

### 2.1. Wireless local area networks

A wireless LAN (WLAN) is a wireless local area network, allowing users to connect directly to a distribution system without interconnecting wires and cables. WLANs utilize spread-spectrum technology based on radio waves whose frequency is much lower than visible light to enable communication between devices in a limited area, also known as the Basic Service Set (BSS).

The primary reasons of the popularity of wireless LANs are their convenience, cost efficiency, and ease of integration with other networks and network components [13].

**Figure 1.**
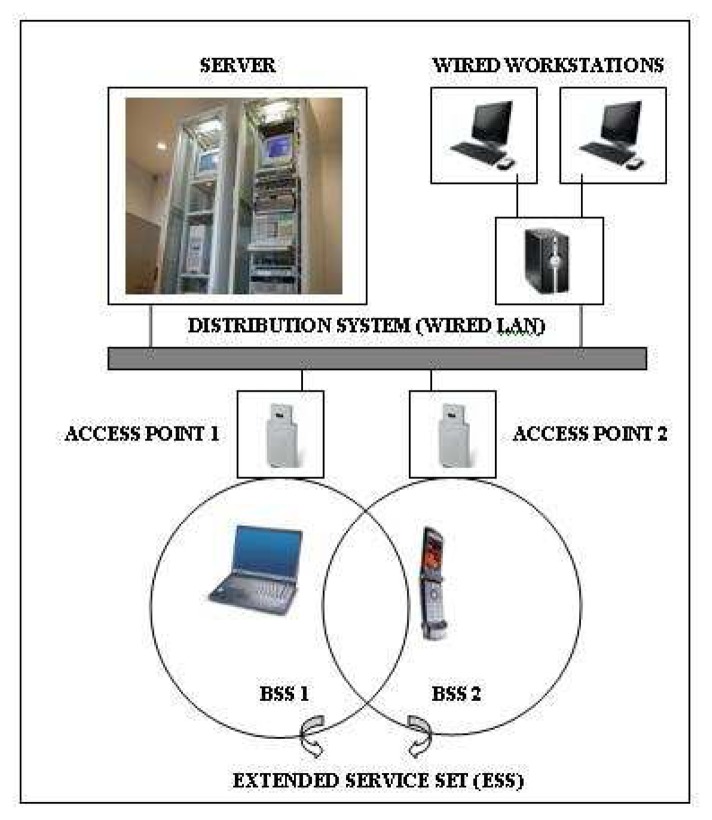
A WLAN Architecture using BSS infrastructure. Adapted from Nichols and Lekkas [13].

Figure 1 illustrates a WLAN Architecture using BSS infrastructure. Server and wired workstations connected to a distribution system called wired LAN and access points are connected to this distribution system. Access points provide BSS communication areas between devices. All BSS areas constitute an Extended Service Set (ESS). The connections to the end-users in Wireless LANs are established via an air interface and the communication is maintained by an electromagnetic coverage area through WLAN Access Point (AP).

With the rapid growth of wireless communications, cell sizes are getting smaller and site-specific propagation information is needed for the design of mobile systems. Coverage is simply the distance that a wireless network can transmit data at a given data rate subject to the regulations in its frequency band and the standard under which it operates. Indoor electromagnetic coverage is a primary consideration in the implementation of indoor wireless networks especially in the frequency range from 500 MHz to 5 GHz. Indoor coverage is important for WLANs where the indoor coverage directly impacts the critical capacity and cost. WLANs are mostly implemented in indoor environments and a circular coverage is expected, but the pattern of the coverage can usually be affected in a destructive or a constructive way. Thus, the coverage area the range and the radiation pattern of a WLAN communication system probably differ from the theoretical prediction approach [14, 15]. An indoor environment is usually very changeable, due to moving people, doors, windows, lifts, furniture and equipment. Indoor signal measurement and prediction are still therefore a kind of a ghost story [1].

The mechanisms behind electromagnetic wave propagation are diverse, but can generally be attributed to reflection, penetration, diffraction and scattering. Most mobile wireless communication systems operate in areas where there is no line of sight path between transmitter and receiver. Due to multiple reflections from various objects, the electromagnetic waves travel along different paths of varying lengths. The interaction between these waves causes multi path fading at a specific location, and the strengths of the waves decrease as the distance between the transmitter and receiver increases.

Aside from direct application in propagation modeling, GIS functionality is clearly essential in preparing data for the construction of a propagation specialized database. Many models require detailed geographic information i.e. location and material parameters [2].

Cisco Aironet 1100 Series Access Point is used for the WLAN communication system at the entrance floor of the T-Block building in Yildiz Technical University. The indoor electromagnetic field measurements and coverage area analysis were implemented according to these access positions. The Access Point has the main following features [25]:

- 2.4 GHz IEEE 802.11g Radio Standard
- Configurable output power up to 100 mW
- 10.4 cm wide; 20.5 cm high; 3.8 cm deep physical dimensions
- Integrated 2.2 dBi dipole antennas
- Up to 54 Mbps date rate for range of 27 m

With the development of science and technology, Wireless LANs, Global System for Mobile communications (GSM), TV-radio transmitters, base stations etc. are used commonly for personal, industrial and commercial aims at every steps of life. The risk factor of electromagnetic pollution for environment and human health has been discussed by many scientists and a lot of research has been done in developed countries. As a result of this, electromagnetic radiation, density and frequency of sources must be under control as described in standards. This study also interrogates the radiation level of WLAN (2.4 GHz) and provides some insight for these areas of research.

### 2.2. Kriging interpolation

Spatial interpolation is a procedure of estimating the values of properties at unsampled locations based on the set of observed values at known locations. A large number of interpolation methods (Inverse distance weighted, Spline, Natural Neighbors, Kriging, etc.) have been developed [16].

It is not possible to measure every point for getting data, therefore, measurement values by interpolation methods are predicted. Interpolation methods must be chosen according to the modeling data type in order to get more accuracy. Besides, adequate number and efficient distribution of measurements provide reliable results.

The Kriging method assumes that the distance or direction between sample points reflects a spatial correlation that can be used to explain variation in the surface. Kriging is a multi-step process; it includes exploratory statistical analysis of the data, variogram modeling, creating the surface, and optionally, exploring a variance surface. Inverse Distance Weighted and Spline are referred to as deterministic interpolation methods because they are directly based on the surrounding measured values or on specified mathematical formulas that determine the smoothness of the resulting surface. A second family of interpolation methods consists of geostatistical methods such as Kriging, which are based on statistical models that include autocorrelation (the statistical relationship among the measured points). Because of this, not only do these techniques have the capability of producing a prediction surface, but they can also provide some measure of the certainty or accuracy of the predictions. There are two important Kriging methods used; Ordinary Kriging and Universal Kriging. Ordinary Kriging is the most general and widely used of the Kriging methods. It assumes the constant mean is unknown. Universal Kriging assumes that there is an overriding trend in the data and it can be modeled by a deterministic function, a polynomial. This polynomial is subtracted from the original measures points, and the autocorrelation is modeled from the random errors. Once the model is fit to the random errors, before making a prediction, the polynomial is added back to the predictions to give you meaningful results [17].

### 2.3. Artificial neural networks in GIS

**Figure 2.**
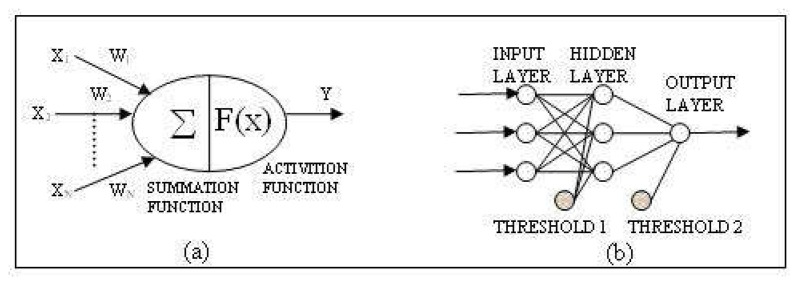
(a) Processing unit or neuron. (b) Architecture of a multi-layer feed-forward network with 3 units in the input layer, 3 units in the hidden layer and 1 unit in the output layer (3-3-1) and threshold units. Adapted from Rigol *et al.* [6].

Artificial Neural Networks (ANNs) are information processing systems that have the ability to implement new information formation and discovery automatically using the mode of learning of human brain and neural biology. ANNs are generally used for classification, prediction, identification, recognition and interpolation problems. The basic processing elements of an ANN are the neurons (units). A neuron has five basic parts. These are; input, weight, summation function, activation function and output as shown in Figure 2a. These units are interconnected by weighted links to form a network. The multi-layer ANN model is typically composed of three parts: input, one or many hidden layers, and an output layer as shown in Figure 2b.

The weights are connections between neurons while the activation functions are linear or non-linear algebraic functions. When a pattern is presented to the network, weights and neurons are adjusted so that a particular output is obtained. Neural networks provide a learning rule for modifying their weights and neurons. Once a neural network is trained to a satisfactory level, it can be used with novel data. Training techniques can either be supervised or unsupervised. Supervised training methods are adapted for interpolation problem [12]. ANNs have recently started to be used for spatial data interpolation in an attempt to overcome some of the limitations presented by more traditional methods [6]. New solutions about spatial interpolation in GIS must be performed by more tools modeling different ANN and need to be discussed about the results.

In general ANN form; a unit in the network sums the weighted inputs from the links feeding into it. The summation function is:

(1) $$NET_{j}^{a}=\sum_{k=1}^{n}A_{kj}C_{k}^{j}$$

where *A_kj_* and $C_{k}^{j}$ are matrixes of weights and outputs respectively.

The activation function applied to both hidden and output layers such as a non-linear Sigmoid Function is shown below.

(2) $$C_{j}^{a}=\frac{1}{1+e^{-(NET_{j}^{a}+\beta_{j}^{a})}}$$

where $\beta_{j}^{a}$ is threshold unit and output of threshold unit is constant and equal to one.

The output is then fed to other units linked to it. In this study, during the training of a feed-forward network the weights of the network are adjusted in a process called back-propagation. As the algorithm's name implies, the errors (and therefore the learning) propagate backwards from the output nodes to the inner nodes so as to minimize the error which is difference between the output of the net and the desired output. So technically speaking, back-propagation is used to calculate the gradient of the error of the network with respect to the network's modifiable weights. This gradient is almost always then used in a simple stochastic gradient descent algorithm to find weights that minimize the error.

The implementation of an ANN requires three main steps: model and architecture selection, training (also called learning) and independent performance assessment (testing). First the appropriate network model and architecture are selected [6]. In order to determine the best network topology, samples chosen for input data, the number of neurons at hidden layer, iterations, learning and momentum rate are changed by several combinations until obtaining an acceptable accuracy. The network having the lowest error is selected. ANN techniques are reviewed in detail by Freeman and Skapura [18] and Bishop [19].

## 3. Methodology

### 3.1. The measurements

The study area is the entrance floor of Yildiz Technical University's T-Block building at the Besiktas Campus in Istanbul, Turkey. In order to produce a map and 3-dimensional model of the study area, the T-Block building, observation points and details inside the building were surveyed by geodetic methods and a Totalstation was used. All details of T-Block building; classrooms, corridors, stairs, doors, columns, central heating radiators and access points of WLAN (2.4 GHz) were surveyed.

In addition to this, the electromagnetic field values of 1085 observation points inside the T-Block building were measured. Electromagnetic field measurements, which are used for analyzing and predicting the electromagnetic coverage area, were performed at the entrance floor of T-Block building. In order to symmetrically cover the floor, 217 straight points were chosen in the corridor, which has an area of 150 square meters. To analyze the 3-dimensional electromagnetic coverage, the measurements were repeated at five different height levels at 50 cm, 100 cm, 140 cm, 215 cm and 290 cm height from the floor. Electromagnetic measurements were performed with an EMR-300 radiometer at every single point and the device was fixed at a constant position by using a tripod. The EMR-300 Radiometer is a versatile system for measuring electromagnetic fields. After setting the measurement system, the device turned on for at least three minutes at a given single position and waited for finding the average electromagnetic field values in units of Volt/meter (V/m). For every single point the same measurement procedure was repeated. The investigated Cisco Aironet 1100 Series Access Point is nearly at the top center of the corridor and attached to the outside walls of the classrooms. It is at 290 cm from the floor.

### 3.2. Data preparation and use of geographic information systems

A GIS is a computer system capable of capturing, storing, analyzing and displaying geographically referenced information; that is, data identified according to location. The power of GIS comes from the ability to relate different information in a spatial context and to reach a conclusion about this relationship [20]. A GIS is built around an integrated database that supports the functions of all units that need spatial processing or even mapping [21]. Although numerous definitions of geographic information and GIS can be found in the literature, all focus on the concept of geo-referencing the association of locations in the geographic domain with the properties of those locations [22].

In this study, the proposed GIS includes the maps of the study area, 3-dimensional model of the electromagnetic propagation environment, electromagnetic field values of observation points, electromagnetic coverage represented by interpolation patterns, the number, position and transmitter power of access points and information about electromagnetic pollution. ArcGIS, an integrated collection of GIS software products, was used for this study. ArcGIS desktop provides a collection of software products that create, edit, import, map, query, analyze, and publish geographic information.

**Figure 3.**
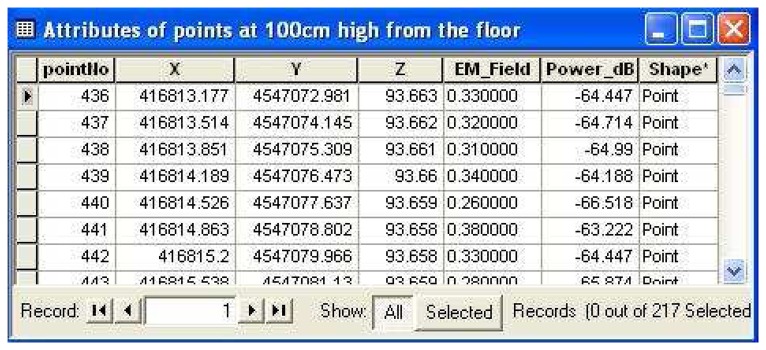
Attribute table of the points at 100 cm high from the floor; electromagnetic field values (V/m) are in the column “EM_Field” and power values (dB) are in the column “Power_dB”.

The T-Block building and observation points were mapped based on the national coordinate system. A personal geodatabase was performed and electromagnetic field value data were stored in that database. Figure 3 illustrates an attribute table of the points at 100 cm high from the floor; electromagnetic field values in units of Volt/meter (V/m) and electromagnetic power values in units of decibel (dB) calculated by equation (3).

The power received at distance d can be calculated in terms of power flux density and effective aperture of the receiving antenna. Relation between electric field and received power is given by: [23].

(3) $$P{\left(d\right)}_{dB}=10\log\left(\frac{|E{\left(d\right)}^{2}|G_{r}\lambda^{2}}{480\pi^{2}}\right)$$

where Gr is the receiver antenna gain, and *λ*= *c* / *f*_0_ is the wavelength, *c* = 3.10^8^*m* / *s* is the velocity of light and *f*_0_ = 2.4*GHz* is the operating frequency of the wireless transmitter. In this calculation receiver antenna gain is assumed as unity.

In addition, the material parameters (iron, steel, wood, glass, concrete etc.) used in the construction of T-Block were stored into the database in order to analyze reflection, penetration, diffraction and scattering effects. All the details are determined and transferred into the GIS in order to present data about propagation environment. Thus, the proposed system provides to make queries and analysis and utilize the results.

**Figure 4.**
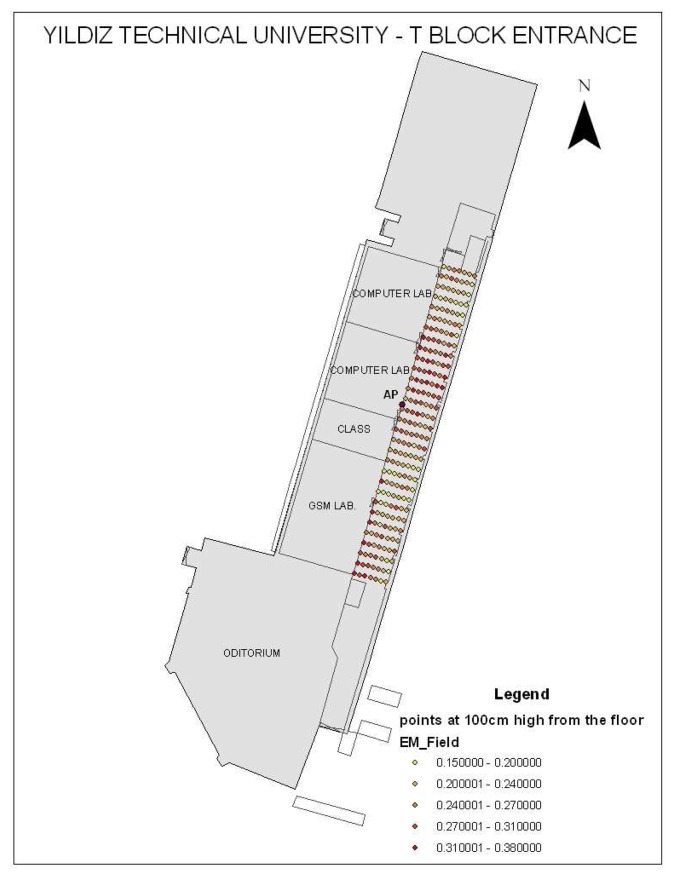
T-Block entrance floor map, access point (AP), observation points and electromagnetic field values (V/m) at 100 cm from the floor.

A map of T-Block entrance floor, access point (AP), electromagnetic measurement points (observation points) in the corridor at 100 cm high from the floor and their values classified by five different colors is shown in Figure 4. The colors range from 0.15 V/m to 0.38 V/m; yellow colors are lower electromagnetic field values and the red colors are higher values. Access point is nearly at the top center of the corridor wall and 290 cm from the floor and provides wireless communication.

**Figure 5.**
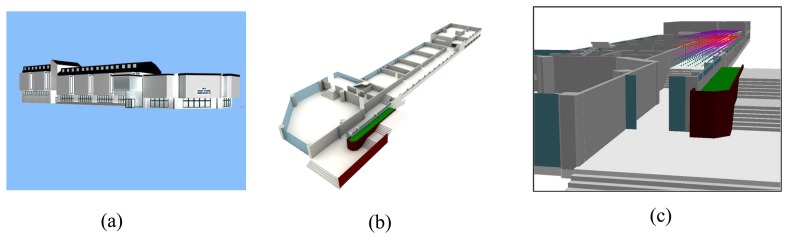
(a) T-Block building. (b) T-Block entrance floor. (c) Observation points along the corridor at 5 different height levels.

The propagation environment was also represented as 3-dimensional. The T-Block building was modeled in order to provide 3-dimensional viewing and this presentation as shown in Figure 5. 1085 observation points at five different height levels were added into the 3D model, as shown in Figure 5c.

### 3.3. Application of Kriging interpolation

The measurement points are separately interpolated for five different height levels (50, 100, 140, 215 and 290 cm from the floor) by the Kriging method. The Kriging interpolation tool is under the “Interpolate to Raster” menu in the 3D Analyst module of ArcGIS. The Kriging tool uses two functions for selecting the neighbor points in interpolation; these are *fixed* and *variable* types. In addition to this, two Kriging methods; *ordinary* and *universal* and semivariogram models; *spherical, circular, exponential, Gaussian* and *linear* are chosen according to the data type and distribution. Properties of Kriging interpolation tool are reviewed in detail by Bratt and Booth [17].

**Figure 6.**
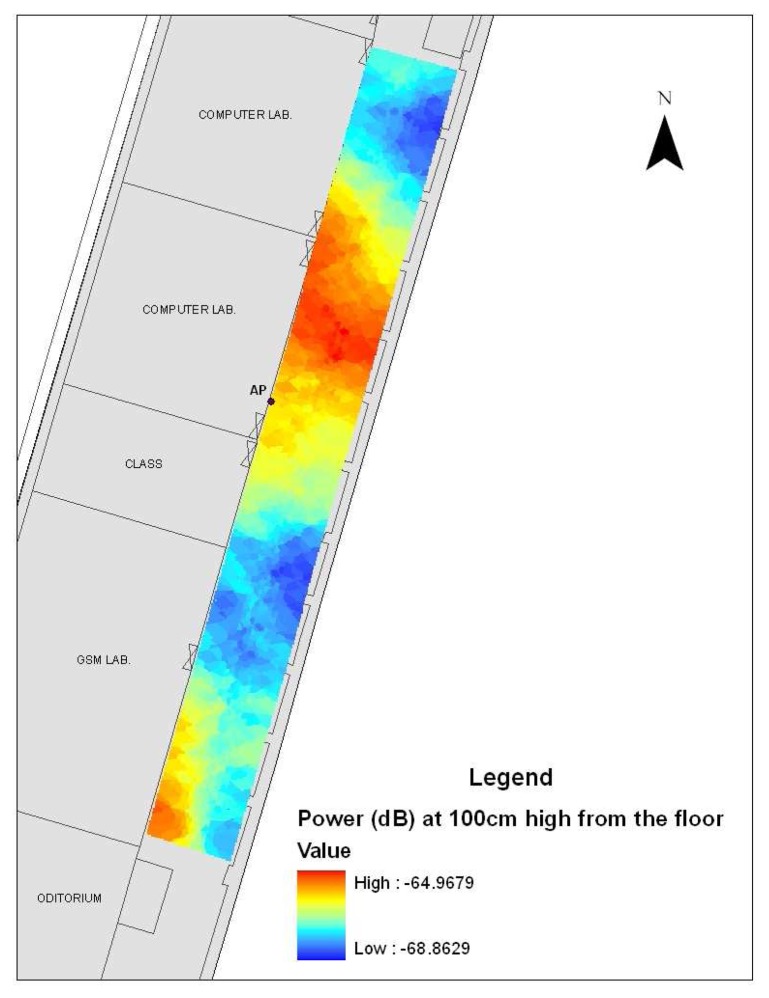
Kriging interpolation pattern of the electromagnetic power values (dB) at 100 cm from the floor.

**Figure 7.**
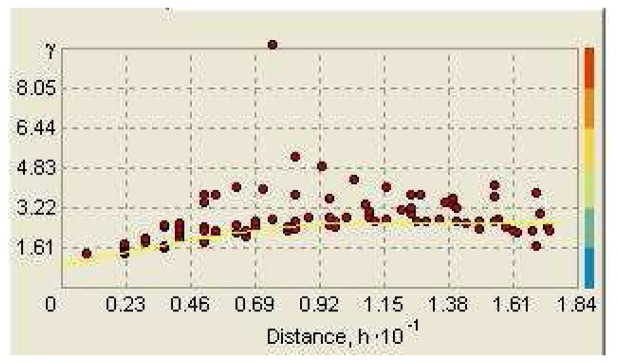
The graph of spherical semivariogram model.

Figure 6 illustrates the Kriging interpolation pattern of the power values (dB) of observation points at 100 cm height from the floor and position of the access point (AP). The colors range from -68.86 dB to -64.97 dB; blue colors are lower electromagnetic power values and the red colors are higher values. For this study, ordinary Kriging with spherical semivariogram model was chosen. The graph of the spherical semivariogram model (Major range: 11.129; Partial Sill: 1.7416; Nugget: 0.89699), along with the experimental points at 100 cm from the floor is shown in Figure 7.

### 3.4. Programming a neural networks tool in GIS for spatial interpolation

In this study, a multilayer feed-forward back-propagation ANN module was integrated in GIS by programming ArcObjects with Visual Basic to interpolate the indoor electromagnetic field measurements. Indoor radio wave propagation was modeled with 3 dimensional GIS dataset in order to analyze the electromagnetic coverage pattern by the neural network interface. Different from Kriging interpolation, all measurements at five different height levels join to the ANN interpolation together and users can query for every altitude.

ArcObjects is a set of programmable objects and Visual Basic is an object-oriented programming language and comes included with ArcGIS. ArcObjects are a set of computer objects specifically designed for programming with applications. ArcObjects can be used to program other applications such as toolbars, buttons, tools, menus and commands as well [24].

#### 3.4.1. The topology of multilayer feed-forward back-propagation artificial neural network

**Figure 8.**
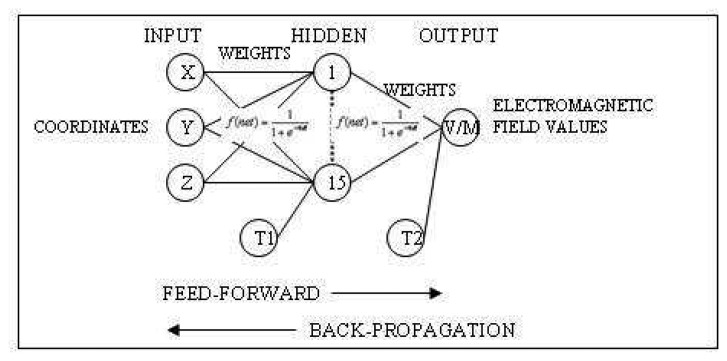
The topology of artificial neural networks for spatial interpolation of electromagnetic field values; x-y-z are the input coordinates, T1 and T2 are threshold matrixes, V/M is electromagnetic field value and 15 neurons in the hidden layer.

In this study; an artificial neural network (ANN), which is composed of one input layer with (k=3) neurons representing x-y-z coordinates, one hidden layer with (j=15) neurons and (m=1) output layer with a single neuron representing the electromagnetic field value (V/m) were used. Besides, threshold matrixes were applied through the hidden and output layers. Back Propagation training algorithm was implemented on the feed-forward network. The x-y-z coordinates were used as input data and they were reduced by replacing a point to the origin (0-0-0 values) of the coordinate system in order to mean the transfer function. Then the other measurement points were referenced to that point. The transfer function applied to both hidden and output layers was a non-linear Sigmoid Function. Figure 8 represents the topology of neural network.

The 1085 measurement points were separated into two groups as training data (672 points) and test data (413 points), respectively. Firstly, the neural network was trained by the input of 672 points and back-propagation calculation performed for every training point in order to distribute the errors to weights. After 200 iterations the final updated weight matrix was found. 413 input points were tested by the updated network with optimized weight matrixes and the average error and accuracy of the neural network was calculated.

The accuracy values of the selected 3-15-1 ANN model and some of the other neural networks trained for spatial interpolation are shown in Table 1. The accuracy of the results was determined by the Root Mean Square Error (RMSE) and the Mean Absolute Error (MAE). RMSE, square root of mean squared predicted electromagnetic power minus observed electromagnetic power and MAE, mean after all errors made positive.

#### 3.4.2. Programming the application with Visual Basic in GIS; the ANN interface

Proposed neural networks (3-15-1 network) module for spatial interpolation was programmed with the visual Basic Editor. The neural network interface provides the electromagnetic field and power values with adequate accuracy for every coordinates (x-y-z) input in the boundary of measurement area.

**Figure 9.**
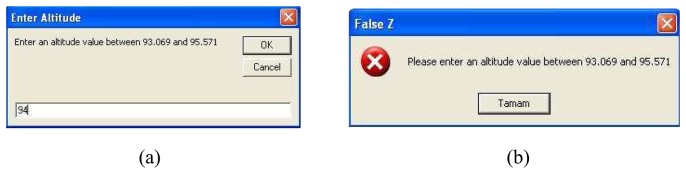
**(a)** Input box for entering Z coordinate. **(b)** Warning message.

There are two parts in ANN module programmed in GIS. These are the “Enter xyz” and “Run ANN” buttons. The “Enter xyz” button is used for selecting the points from the map and assigning the point coordinates to ANN. When a point is selected from the map screen by the cursor, x and y coordinates are assigned automatically. Hence the z coordinate can be entered by using an input box called “Enter Altitude”, as shown in Figure 9a. Because altitude value cannot be selected by the cursor from the map screen 2-dimensional view. The user can write an altitude value between 93.069 and 95.571 which are the measurement intervals. If the user enters a value out of this interval, it gives a warning message as shown in Figure 9b.

**Figure 10.**
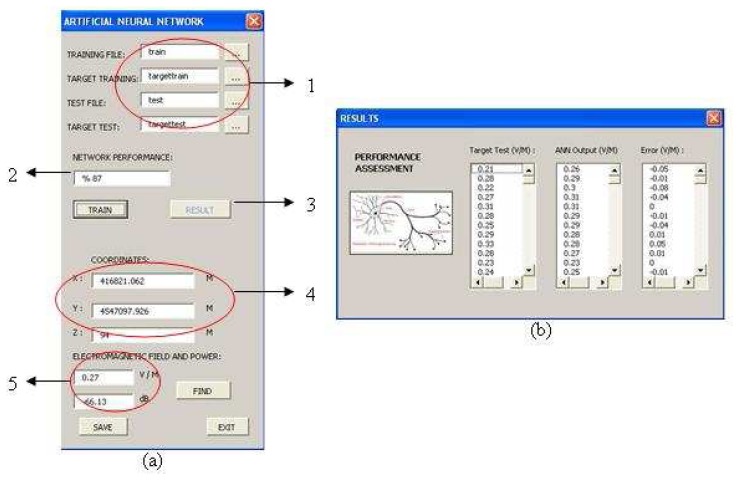
(a) Artificial neural network tool for spatial interpolation of electromagnetic field values. (b) The performance assessment of test results with error values.

After entering coordinates of the point, ANN interpolation interface is executed by “Run ANN” button. Figure 10 illustrates the ANN interface and performance assessment of the test results by error values (V/m).

The user interface is formed by these following sections as shown in Figure 10a:

1. The files about 672 training points, 413 test points and observed electromagnetic field values are added from the computer.
2. After training, 413 input points are tested by the updated network with optimized weight matrixes and the average error and accuracy of the neural network is calculated by equation (4) (network performance).
3. “Result” button executes another interface as shown in Figure 10b. It is used for comparing between ANN outputs and observed (expected) values of electromagnetic field in test data.
4. Coordinates of the points are added from the map screen mentioned before and it can be rearranged in XYZ boxes.
5. The electromagnetic field (V/m) and power (dB) values are predicted.

The performance rate of ANN (3-15-1 network) is calculated by;

(4) $$P=\frac{D}{T}100$$

where *D* is the number of accurate predictions of ANN output comparing with observed (expected) values of electromagnetic field in test data and *T* is the number of test points (413). As a result, expected accuracy of the network is almost between 85% and 90% performance and the error result can be accepted for interpolation of electric field values and coverage prediction.

#### 3.4.3. ANN interpolation pattern

**Figure 11.**
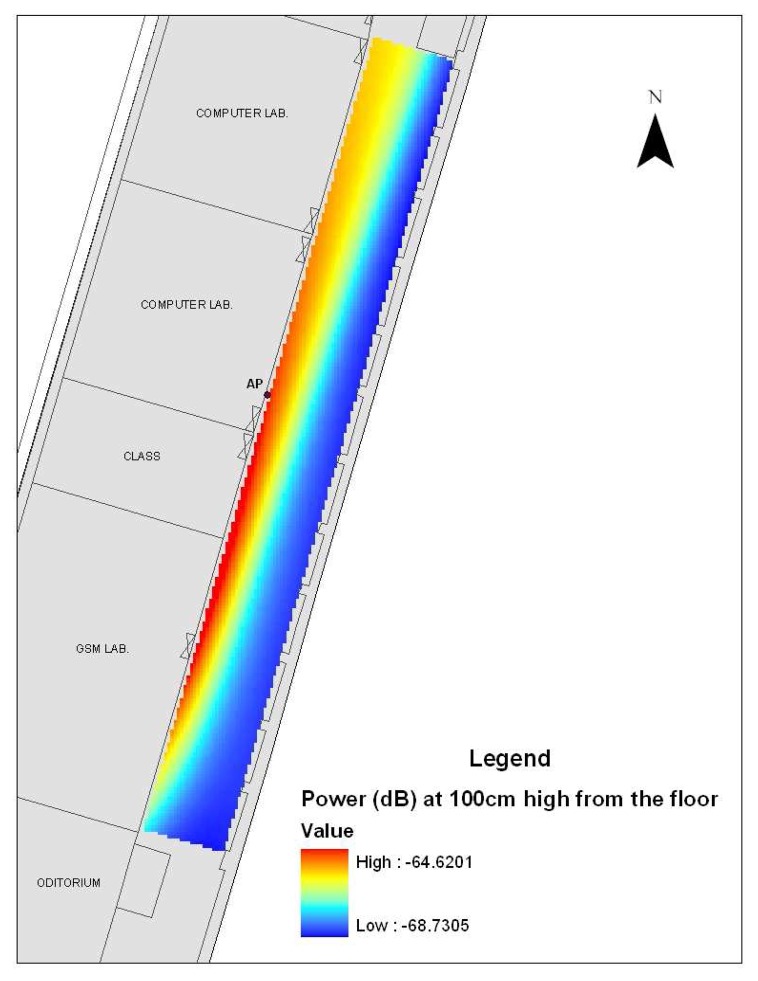
ANN prediction of received power (dB) values at the 100 cm height level.

A multilayer feed-forward back-propagation ANN (3-15-1 network) interpolation pattern of the observation points at 100 cm from the floor and position of the access point (AP) are shown in Figure 11. The colors range from -68.73 dB to -64.62 dB; blue colors are lower electromagnetic power values and the red colors are higher values.

## 4. Results and Discussions

### 4.1. Coverage results

The Neural Network is finally formed with the optimized weight matrixes and these matrixes are set to the feed-forward network. After setting the final neural network, the WLAN coverage was analyzed for 100 cm altitude level which represents the usual height of a WLAN receiver. The coordinate values (x-y-z) defining the 100cm level were applied to the input nodes of the network and the predicted electric field values were given by the output node. The corresponding outputs of the input coordinate values were converted to the units of received power (dB;, and then they were sketched as Figure 11 representing the cross-section radiation pattern of the WLAN access point.

The predicted coverage figure shows a linear propagation varying between -68.73 dB and -64.62 dB power values. In several attempts, it was noticed that various types of WLAN adapters could access to the system even below the -70 dB threshold. Thus, in a range of 27 m, the radiating WLAN access point can almost cover the whole corridor to satisfy up to a 54 Mbps communication with a IEEE 802.11g compliant WLAN Adapter [25]. However, actual throughput may vary based upon numerous environmental factors and the efficient communication data rate cannot be achieved for low power level points as shown in Figure 11. Moreover, this electromagnetic coverage does not lead to an electromagnetic pollution due to the low power levels [26].

### 4.2. Comparison between ANN prediction and Kriging interpolation method

The electromagnetic coverage in the propagation environment now can be modeled by both ANN prediction and Kriging interpolation method. The network architecture selected was in this case 3-15-1, that is three input nodes, 15 hidden nodes and one output node. The Kriging interpolation pattern in Figure 6 shows that the electromagnetic power values of WLAN (2.4 GHz) changes between -68.86 dB and -64.97 dB at 100 cm from the floor and there are sudden changes in radio wave propagation due to the environmental parameters (reflection, penetration, diffraction and scattering). Hence, the ANN prediction pattern in Figure 11 shows a linear propagation varying between -68.73 dB and -64.62 dB power values at the same height. It seems that the feed-forward back-propagation ANN (3-15-1 network) for spatial interpolation makes a generalization according to the learning of network. However, Kriging catches the sudden changes of electromagnetic power distribution.

The predictive power of each of the two interpolation models was compared using Root Mean Square Error (RMSE) and The Mean Absolute Error (MAE). RMSE, square root of mean squared predicted electromagnetic power minus observed electromagnetic power and MAE, mean after all errors made positive. Table 2 shows the RMSE and MAE of the fully trained 3-15-1 network prediction and Kriging interpolation of electromagnetic power values of 1085 observed points and as a result, Kriging interpolation is more accurate than ANN interpolation of electromagnetic field measurements. An advantage of ANN module programmed in GIS is that ANN prediction uses a Back-propagation algorithm, updating itself by optimizing the weight matrixes to enable a three-dimensional (3D) query.

## 5. Conclusions

In this study, a multilayer feed-forward back-propagation neural network was developed to interpolate the electromagnetic field measurements by programming a tool with Visual Basic in GIS and coverage prediction was investigated. The comparison of the selected ANN and Kriging was represented by adjusting procedures. The feed-forward back-propagation ANN provides adequate accuracy for spatial interpolation. However, Kriging interpolation is more accurate than ANN predictions. Concerning the interpolation patterns, ANN (3-15-1), which is composed of one input layer with (k=3) neurons representing x-y-z coordinates, one hidden layer with (j=15) neurons and (m=1) output layer with a single neuron representing the electromagnetic field value (V/m), generalized the data interpolated. However, Kriging catches the sudden changes of electromagnetic power distribution. Expected accuracy of the neural network is almost between 85% and 90% performance and the error result can be accepted for interpolation of electromagnetic field values and coverage prediction. This paper demonstrated that spatial interpolation with neural networks is a viable technique for electromagnetic power estimation.

The proposed GIS ensures indoor radio wave propagation environment and electromagnetic coverage, 3-dimensional modeling of the study area, the number, position and transmitter power of access points and electromagnetic radiation level. With GIS, it is possible to get information about power of the wireless communication and efficiency of access points. It was noticed that the radiating WLAN access point can almost cover the whole study area and this electromagnetic coverage does not lead to an electromagnetic pollution due to the low power levels.

As a result the proposed GIS system with ANN prediction help a telecom radio frequency designer making queries about the current electromagnetic coverage and pollution analysis in a given propagation environment and determining the communication signal quality. Future research on a number of open issues; the other ANN such as Hopfield networks can be developed for spatial interpolation as a tool in GIS and the electromagnetic coverage of GSM, TV-radio transmitters, base stations and their effects to the human health in cities can be analyzed with GIS using ANN.

## Figures and Tables

**Table 1.** The comparison of ANN models for spatial interpolation of observed points at 100 cm from the floor. MAE, mean after all errors made positive and RMSE, square root of mean squared predicted electromagnetic power minus observed electromagnetic power.

**Neural nets**	**Learning rate**	**Momentum rate**	**Iterations**	**RMSE (dB)**	**MAE (dB)**
3-15-1	0.2	0.4	200	1.51	1.16
3-15-1	0.6	0.7	50	2.82	2.40
3-6-1	0.2	0.4	200	1.83	1.47
3-10-1	0.2	0.4	100	3.21	2.80

**Table 2.** Performance of the selected 3-15-1 network and Kriging interpolation of observed points at 50 cm, 100 cm, 140 cm, 215 cm and 290 cm from the floor. MAE, mean after all errors made positive and RMSE, square root of mean squared predicted electromagnetic power minus observed electromagnetic power.

**Data set**	**Interpolation method**	**RMSE (dB)**	**MAE (dB)**
50 cm height	ANN	1.48	1.13
	Kriging	1.02	0.78

100 cm height	ANN	1.51	1.16
	Kriging	1.04	0.80

140 cm height	ANN	1.52	1.17
	Kriging	1.05	0.81

215 cm height	ANN	1.55	1.22
	Kriging	1.07	0.85

290 cm height	ANN	1.56	1.24
	Kriging	1.08	0.87
